# The Use of SPME-GC-MS IR and Raman Techniques for Botanical and Geographical Authentication and Detection of Adulteration of Honey

**DOI:** 10.3390/foods10071671

**Published:** 2021-07-20

**Authors:** Nefeli Sofia Sotiropoulou, Marinos Xagoraris, Panagiota Kyriaki Revelou, Eleftheria Kaparakou, Charalabos Kanakis, Christos Pappas, Petros Tarantilis

**Affiliations:** Laboratory of Chemistry, Department of Food Science and Human Nutrition, School of Food and Nutrition Sciences, Agricultural University of Athens, Iera Odos 75, 11855 Athens, Greece; nefsot@aua.gr (N.S.S.); mxagor@yahoo.gr (M.X.); p.revelou@aua.gr (P.K.R.); el.kaparakou@gmail.com (E.K.); chkanakis@aua.gr (C.K.); chrispap@aua.gr (C.P.)

**Keywords:** honey authentication, SPME, GC-MS, IR, Raman, chemometrics, botanical and geographical origin, adulteration

## Abstract

The aim of this review is to describe the chromatographic, spectrometric, and spectroscopic techniques applied to honey for the determination of botanical and geographical origin and detection of adulteration. Based on the volatile profile of honey and using Solid Phase microextraction-Gas chromatography-Mass spectrometry (SPME-GC-MS) analytical technique, botanical and geographical characterization of honey can be successfully determined. In addition, the use of vibrational spectroscopic techniques, in particular, infrared (IR) and Raman spectroscopy, are discussed as a tool for the detection of honey adulteration and verification of its botanical and geographical origin. Manipulation of the obtained data regarding all the above-mentioned techniques was performed using chemometric analysis. This article reviews the literature between 2007 and 2020.

## 1. Introduction

Honey is a sweet and viscid natural product produced by bees (*Apis mellifera* L.) either from the nectar of flowers (blossom honey) or from secretions of the living parts of plants other than flowers or is a product of excretions of plant-sucking insects (honeydew honey) [1,2]. It is characterized as a natural sweetener and its consumption is increasing worldwide due to its important role in the human diet, as it is endowed with many beneficial health properties [3,4]. It is estimated that more than 1.2 million tons of honey are produced per year; thus, it is characterized by a high economic importance [5]. The nutritional and economic value of honey is due to its unique composition. Honey contains sugars, water, proteins, organic acids, vitamins, minerals, pigments, phenolic and volatile compounds, and some solid particles [6,7].

The composition of honey depends on collection season, climate conditions, proximity to the forest, characteristics of soil which determine melliferous flora, method of storage, processing, and practices of beekeeping, and even interactions between chemical compounds and enzymes [3,8,9]. However, the aroma and taste of honey, owed to the volatile compounds, are dependent mainly on the botanical and floral origin of honey [9,10,11,12].

Honey authenticity concerns its origin and the mode of its production, which is important both for consumers and producers [7,13]. The authentication of honey is also significant for commercial and health related reasons. Honey gains worldwide popularity among health-conscious consumers and also by consumers who demonstrate great interest in the origin and quality of the product [3,14,15]. Therefore, honey labelling in order to avoid unfair competition and adulteration has become a mandatory demand [16,17].

Nowadays, botanical and geographical originality of honey is a major concern among authorities in order to ensure its quality and authenticity, by imposing specific standards that allow honey to be competitive in the market [3,16]. Traditionally, identification of botanical and geographical origin of honey is performed by melisopalinological analysis [18,19]. This analysis is a time and cost-consuming process which cannot ensure reliable characterization of the honey floral source since it strongly depends on the capability of the analyzer [20,21]. Therefore, it is important to complement this analysis with other, more analytical techniques (physicochemical, organoleptic, chromatographic etc.), or replace it with them. During the past decades, several studies focused on gas chromatography (GC) in order to determine the volatile profile of honey [3,9,11,20,22,23,24,25,26]. The characteristic chemical fingerprint generated by volatile compounds is of major importance regarding consumers’ choice since it provides information about the botanical and geographical origin of honey [27].

Gas chromatography with mass spectrometry (GC-MS) is suggested as a convenient, appropriate technique for the determination of volatile compounds with high precision [28,29,30,31,32,33,34,35,36]. GC-MS provides the separation of volatile components of honey sample and then the necessary information for their identification [2]. Various techniques are used to collect the volatile fraction from a sample, among them Solid Phase microextraction (SPME) has been reported as the most preferable [32,37,38]. SPME is adopted since it is rapid, easy, and effective, and can also be applied to a small sample amount [36,38,39,40,41,42,43,44,45,46]. SPME is also eco-friendly, cheap, sensitive, selective, versatile, and possibly automated [39,41,43]. The chemical profile of honey obtained after GC-MS analysis is usually complex. Consequently, its interpretation requires the use of specific methods in order to simplify results and to give a final report which facilitates honey classification according to its geographical and botanical origin. In this regard, chemometrics is considered a valuable tool of data analysis, widely used by many researchers [47,48].

Another main topic concerning the beekeeping sector, the honey industry, and researchers is the adulteration of honey. According to European Union regulations, the addition or removal of any kind of honey substance is illegal [49]. Honey adulteration is achieved by adding lower quality honey and artificial adulterants [50]. Honey’s health benefits, and its unique flavor and aroma make it more expensive in comparison to other sweeteners. Therefore, in an attempt to reduce production costs and simultaneously increase profit, honey is a product usually subjected to adulteration [9,15,51]. Starch and inverted syrup fed to bees, addition of sugars such as high fructose, glucose, and saccharose syrups, and low-quality honey added to high-priced honey are considered the most common ways of honey adulteration [15,52]. Honey adulteration can occur in any step of production or processing. It is also difficult to detect due to the fact that the adulterated honey is similar to the pure one [53]. Moreover, the classical methods that certify honey quality, such as physicochemical analyses, are incapable of detecting adulteration accurately. Thus, it is essential to develop and adopt a new process for honey quality control. For the aforementioned reasons, many analytical techniques have been applied, characterized by high effectiveness, accuracy, and sensitivity for the detection of honey adulteration [9].

In recent years, vibrational molecular spectroscopy techniques such as infrared (IR) and Raman are used to identify and quantify the chemical composition of various food products with flexibility, efficiency, and low cost [54]. These techniques also provide an easy, reliable, environmentally friendly, non-destructive, and prompt way for honey quality control. In most cases, there is a combination of spectroscopic techniques and chemometrics allowing the visualization and better classification of the samples [16,50,51,55,56,57,58,59]. Despite the fact that advanced and accurate analytical techniques have been performed for honey authentication, there are studies that focus on the development of an authentication model using simple physical and chemical parameters or spectral data [60,61,62]. Therefore, this quest has led to the application of advantageous spectroscopic techniques (IR, Raman) in order to develop this model for botanical and geographical characterization [9,54,63,64]. The combination of these spectroscopic techniques with chemometrics provides satisfactory discrimination and rapid first-line classification of honey based on its botanical and geographical origin [9,16,63,65].

In order to analyze these complex data (e.g., spectral-chemical data sets), chemometrics methods are required, as mentioned before. Chemometric tools are applied in targeted and non-targeted approaches for the identification of food adulteration or verification of their botanical or geographical origin [18,53,55,66,67,68,69,70]. The most common unsupervised methods, used for exploratory analysis in food authentication, include honey authentication, are principal component analysis (PCA) and hierarchical clustering analysis (HCA) [28,59,71,72,73,74,75]. For discrimination and classification purposes, linear discriminant analysis (LDA), partial least squares discriminant analysis (PLS-DA), orthogonal partial least squares discriminant analysis (OPLS-DA), k-nearest neighbor (k-NN), principal component regression (PCR), multiple linear regression (MLR), or partial least squares regression (PLS-R) were commonly performed as supervised methods [28,57,72,73,74,76,77]. For the botanical and geographical characterization of honey, most of the aforementioned chemometric tools have been reported such as PCA, HCA, LDA, and PLS-R, either combined with GC-MS [47,48,77,78,79] or with IR and Raman spectroscopy [9,16,54,56,63,80]. In the case of honey adulteration, the same statistical techniques as above were used combined with spectroscopic analysis (IR and Raman) [50,55,59,64].

The aim of this work was to present a review (period of 2007–2020) of SPME-GC-MS and spectroscopic techniques in combination with chemometrics for honey authentication. In addition, spectroscopic techniques (IR, Raman) combined with chemometric analysis for the investigation of honey adulteration are briefly discussed.

## 2. Honey Volatile Compounds Analysis Using SPME-GC-MS

Honey aroma depends on qualitative and quantitative composition of the volatile compounds; it is one of its most important properties as mentioned before and is considered a significant topic of study. The volatile profile of honey could reveal its botanical and geographical origin; thus, a false characterization can be avoided. Since botanical and geographical classification of honey is an important issue, chromatographic analytical techniques have been developed aimed at its chemical characterization and consequently its classification.

Many extraction methods that have been used to collect honey volatile compounds, combined with GC-MS analysis in order to provide information for honey authenticity [3,9,10,22,81], require consumables, solvents, and a lot of time [41]. Particularly, the solvent extraction has been employed for honey characterization, due to its simplicity and to the fact that is applied without heat. However, this method can solubilize also non-volatile compounds and the solvents can con-elude with analytes [41,82]. Moreover, for the isolation of honey’s volatile compounds, simultaneous steam distillation-extraction has been reported, with the intention of avoiding sugar interference. The main disadvantage of this method is the presence of non-characteristic compounds in honey samples due to exposure to heat [22,41]. In addition, another commonly used method to extract the volatile compounds of honey is purge and trap thermal desorption, providing high sensitivity for fractionation of high-volatility compounds, the absence of extended heating times, and the reproducibility associated with a totally automated system [11,12,18,23,24,25,26,83]. However, this method requires specific and expensive devices [41].

On the other hand, SPME sampling technique is solvent-free, inexpensive, rapid, and simple. It is ideal for the collection of honey volatile compounds since it provides high sensitivity along with effectiveness and requires a small amount of untreated sample [38,41]. However, the adsorption selectivity of the fiber and its discrimination between components of must be considered for quantitative determination of volatile compounds [84].

### 2.1. Botanical Characterization of Honey by SPME-GC-MS

Volatile compounds of honey are related to the floral origin and could be used as biomarkers. SPME followed by GC-MS for determining the volatile profile of honey are used as a tool for the botanical characterization of several different types of honeys [8,11,14,21,27,37,85,86,87,88].

Thus, in unifloral honeys (linden, rape, acacia, and sunflower) from Romania, 98 volatile compounds were detected and classified in seven main classes. Commonly detected compounds in all tested honeys were *β*-damascenone, *cis*-linalool oxide, hotrienol, *p*-cuminalaldehyde, nonanoic acid, phenylethyl alcohol, benzyl alcohol, and benzaldehyde. These compounds can be considered as specific markers for each type of honey. However, in the case of acacia honey, the new specific markers were 8-hydroxylinalool, 2-furfural-aldehyde, 2-hexen-1-ol, 2-hydroxycyclopent-2-en-1-one, 2-phenylethyl isothiocyanate, 2-phenylpropenal, 5-hydroxymethylfurfural, decanal, dimethyl palmitamine, hotreniol, lilac aldehyde C, lilac aldehyde D, linalool oxide, myrtenal, octanoic acid, oleic acid, and pinocarvone. On the other hand, marker compounds for sunflower honey were 3-furfural aldehyde, (3,3-dimethylcyclohexylidene) acetaldehyde, 1,3,3-trimethylcyclohex-1-ene-4-carboxaldehyde, *p*-menthan-3-one, endo-borneol, menthol, myrtenol, verbenone, isopiperitone, *p*-cymen-7-ol, eugenol, *β*-calarene, *cis*-linalool oxide (furanoid), and hotrienol. The specific markers for linden honey were the compounds 6-allyl-o-cresol, allylphenylsulfide, butanoic acid, 3,6-dimethyl-4,5,6,7-tetrahydro-1-benzofuran, 1-methyl-4-(1-methylpropyl)-benzene, *trans*-dihydrocarvone, *o*-methylacetophenone, isoneral, isopropyl benzene, geranic acid, sabinene, teresantalol, 2-undecenal, *p*-cymene, and myrtenyl acetate, while rape honey as characterized by the presence of 3-methylpentanol, pentanoic acid, ethyl 2-hydroxy-4-methylbenzoate, *trans*-linalool oxide, *α*,*α*,4-trimethylbenzyl alcohol, lilac alcohol C, 3-phenyl propanol, dihydro-5-propyl-2(3*H*)-furanone, ethyl decanoate, ethyl 3-hydroxytridecanoate, ethyl dodecanoate, 2,2,4-trimethyl-1,3-pentanediol diisobutyrate, ethyl palmitate, ethyl oleate, ethyl benzoate, and 3-methylbutanol [8].

Volatile compounds of unifloral *Thymus capitatus*, *Thymelaea hirsute*, and *Tolpis virgata* honeys from Palestine were determined also by HS-SPME-GC-MS (headspace-solid-phase microextraction-gas chromatography-mass spectrometry). Aldehydes, organic acids, phenols, and alcohols were present in all the honeys. Chemical markers for *Thymus capitatus* honey were 1,3-diphenyl-2-propanone, (3-methylbutyl) benzene, 3,4,5-trimethoxybenzaldehyde, 3,4-dimethoxy benzaldehyde vanilline, and thymol. In the case of *Thymelaea hirsuta* honey, benzene propanol, benzylalcohol, nonanol, hexanol, and 4-methoxyphenol were its characteristic compounds. *Tolpis virgata* honey was characterized by 3,5-dihydroxytoluene and tridecane [86].

Monofloral (rape, caraway, and white clover) and polyfloral honey from Lithuania were studied. The chemical classes of the volatile compounds were the same for all studied samples, but their botanical classification was achieved through qualitative and quantitative differences between these classes. Rape honey was distinguished by high percentage of *p*-cymenene, while a high amount of benzaldehyde characterized the caraway honey [12].

Croatian honey samples of *Paliurus* honey were dominated by nonanal, four isomers of lilac aldehyde, decanal, methyl nonanoate, hexanoic, and 2-ethylhexanoic acids [27].

By performing also SPME-GC-MS, the most important components of Ulmo honey flavor were benzaldehyde, *β*-damascenone, octane, nonanal, 4-methoxybenzaldehyde, isophorone, lyrame, and 4-vinylanisole [14].

The volatile profile of three endemic different blossoms of Brazil were determined. Aromatic aldehydes of juazeiro honey, sulfur compounds and ketones of jurema honey, and volatile acids of velame branco could be considered as markers of “blossom type” origin [21].

Different varieties of popular Polish honeys were studied by Plutowska et al. [37]. Heather honey was characterized by the presence of 3,4,5-trimethylphenol, phenylacetic acid, *β*-damascenone, benzoic acid, and isophorone (3,5,5-trimethyl-2-cyclohexen-1-one), and linden honey by dimethylstyrene. In the case of acacia honey, characteristic compounds were benzaldehyde, nonanal, and phenylacetaldehyde, and for honey-dew honey were 2,3-butanethiol and acetic acid isomers.

Buckwheat honey from Poland was characterized by furfural, 2- and 3-methylbutanoic acid, and 2- and 3-methylbutyraldehyde [26], while 3-methylbutanoic acid also presented at high levels in the buckwheat honeys from Italy and east Europe. Thus, 2-methylbutanal and phenylacetaldehyde could be considered as botanical markers [87].

In rape honey, benzoic acid and its ethyl ester were considered the most characteristic and abundant compounds [8,37]. Other compounds suggested as markers for rape honey include benzoic alcohol [37], 1-pentanol-3-methyl, and 1-butanol-3-methyl [8].

In another study by Špánik et al. [88], selected chiral volatile compounds of acacia, chestnut, linden, rapeseed, orange, and sunflower honeys were determined for botanical characterization using SPME-GC-MS techniques. Specially, rapeseed honey was characterized by the variation in enantiomer ratio of linalool. In cases of acacia and oranges honeys, differentiation was achieved by enantiomer ratios of lilac aldehydes. Finally, a different enantiomer ratio of 4-terpineol was found in sunflower honey.

During the past decade, it has been noted that SPME-GC-MS fingerprinting of honey volatiles combined with chemometrics can be considered as non-time and of high potential combination also for routine analyses of honey for their botanical characterization.

**Figure 1:**
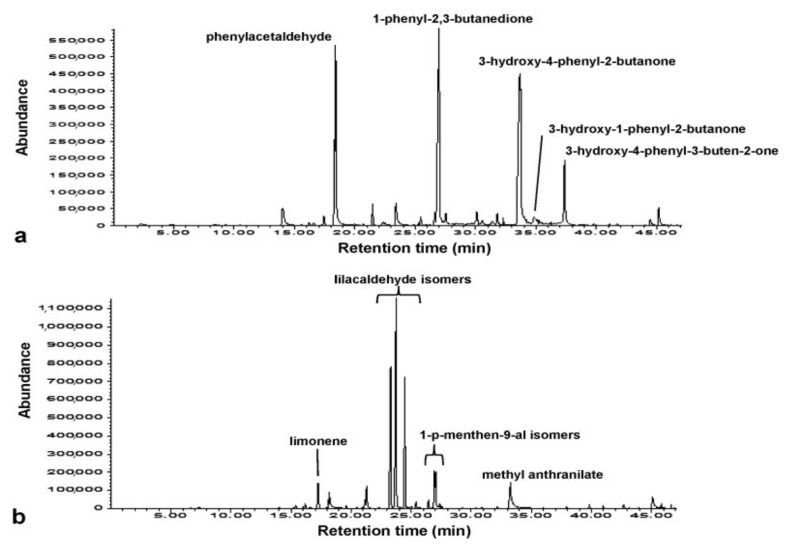
Representative chromatograms of thyme (**a**) and citrus (**b**) honey samples extracted performing HS-SPME. Compounds that serve as biomarkers for their discrimination are indicated. Reprinted with permission from ref. [47]. Copyright 2017 Copyright Elsevier B.V.

Differentiation of citrus and thyme honey produced in Greece was achieved using SPME-GC-MS. Chemometric models, namely principal components analysis (PCA), orthogonal partial least squares-discriminant analysis (OPLS-DA), OPLS- hierarchical cluster analysis (OPLS-HCA), and soft independent modelling of class analogy (SIMCA) were used to analyze results. Lilacaldehyde, limonene, methyl anthranilate, and 1-*p*-menthen-9-al isomers were biomarkers for citrus honey. For thyme honey, phenylacetaldehyde, 3-hydroxy-4-phenyl-2-butanone, 3-hydroxy-1-phenyl-2-butanone, 1-phenyl-2,3-butanedione, and 3-hydroxy-4-phenyl-3-buten-2-one were the compounds’ biomarkers that allowed its discrimination from citrus honey (Figure 1) [47].

A “leave-one-out” cross validation procedure proved the stability of the model concerning the botanical origin of thistle honey from Italy. The volatile compounds detected in all the analyzed thistle honeys were decanal, nonanal, furfural, 3,6-dimethyl-2,3,3*a*,4,5,7*a*-hexahydrobenzofuran, benzaldehyde, hotrienol, *α*-linalool, lilac aldehyde (isomer IV), phenylacetaldehyde, 4-oxoisophorone, benzyl alcohol, 2-phenylethanol, methyl anthranilate, octanoic, and nonanoic acids. This developed model was based on the valuation of the relative amounts of the above compounds. Each thistle honey was removed one at a time from the initial model, then the model was rebuilt, and the honey sample removed was classified as the new model [17].

Corsican chestnut catkins and chestnut grove honeys were also characterized from volatile compounds to provide information for their botanical authenticity, using PCA and cluster analysis (CA) as statistical methods. It was found that the main compounds of chestnut catkins were acetophenone, methyl salicylate, linalool, and nonanal, and 2-aminoacetophenone, acetophenone, benzaldehyde, nonaic and octanoic acids, and 3-furaldehyde were the dominant compounds of chestnut grove honeys [89].

In a study by Karabagias et al. [60], Greek unifloral honeys (thyme, pine, fir, and orange blossom) were characterized according to the botanical origin using headspace (HS)-SPME-GC-MS and Linear Discriminant Analysis (LDA). Results showed that 30 volatile compounds of different classes classified the different unifloral honeys, by achieving a classification rate of 84.0% using the leave one out cross validation method. In another study by Karabagias et al. [77], volatiles for the authentication of monofloral honey coming from different varieties of Greek honey (citrus, fir, pine, and thyme) were determined. Specifically, only nine volatile compounds were used for the botanical classification (*α*-4-dimethyl-3-cyclohexene-1-acetaldehyde, dill ether, acetic acid ethyl ester, octanoic acid ethyl ester, methylanthranilate, 2,2,4,6,6-pentamethyl-heptane, phenylacetaldehyde, cis-linalool oxide and lilac aldehyde (isomer III)) using PCA, CA, LDA, multivariate analysis of variance (MANOVA), stepwise linear discriminant analysis (SLD), and k-nearest neighbor analysis (k-NN).

Thirteen different honey types from four different botanical origins (heather, raspberry, rape, and alder-buckthorn) from Estonia were studied. Agglomerative hierarchical clustering and correspondence analysis showed that none of the identified volatile compounds were determined solely at one blossom honey type. However, in the case of heather honey, isophorone and 2-methylbutyric acid were identified as characteristic compounds [38].

A total of 100 samples from six different varieties (*Acacia nilotica*, *Acacia seyal*, *Ziziphus spina-christi*, *Amaranthus graecizan*, *Eucalyptus* spp., and multifloral) of Sudan were studied. Choosing the twenty most abundant and characteristic volatile compounds and by applying multivariate analysis (HCA, PCA and partial least-squares regression, PLSR), the honey samples were clearly distinguished based on the floral type [79].

Da Costa et al. [21] characterized different monofloral honeys produced in the Brazilian semiarid region based on their volatile profile. It was suggested that linalool for malícia honey, *D*-sylvestrene for chanana honey, rose oxide for algaroba honey, and benzenethanol for angico honey are the markers for botanical sources. This variation was visualized and confirmed by PCA.

Kortesniemi et al. [13] determined the odor active compounds of Finnish honeys such as buckwheat, cloudberry-bog, lingonberry, sweetclover, willowherb, and multifloral honeys, applying SPME-GC-MS-O (olfactometry) and statistical methods (PCA, PLS). They reported that odor active compounds of honeys showed variation in Finnish honeys from different floral sources.

A total of 14 rare lacy phacelia (*Phacelia tanacetifolia* Benth.) honey samples from Poland were analyzed. According to PCA and hierarchical-tree clustering (HTC), it was found that hexan-1-ol and lavender lactone were characteristic biomarkers for authentication of rare lacy phacelia honey [4].

The botanical source of heather honey from northwest of Iberian Peninsula was evaluated by volatile compounds analysis combined with PCA and Spearman’s rank correlation. There were 58 volatile compounds identified, including terpenoids, alcohols, benzene compounds, furan derivatives, and aldehydes. The marker volatile compound of heather honey was hotrienol. Moreover, phenylacetaldehyde and *cis*-linalool oxide were found at high amounts [90]. Table 1 shows a summary of the literature survey based on the volatile compounds of honey as botanical markers.

### 2.2. Geographical Characterization of Honey by SPME-GC-MS

As mentioned before, volatile profile of honey has been proposed as marker also for geographical authentication.

Cioltaus at al. [8] successfully distinguished multifloral honeys from different areas of Transylvania, using SPME-GC-MS technique. The differentiation of volatile profile was obtained between varieties of different native floral sources (mountain, hill, valley, urban). *D*-limonene, isopulegol, 1-octen-3-ol, 1-octen-3-one, lilac aldehyde B, *β*-elemene, and *trans*-calamenene were identified as honey markers for the valley source. Similarly, dimethyl sulphide, 3-hydroxy-butanal, isodihydro lavandulyl aldehyde, octanoic acid, myrtenol, and *2*,4-di-tert-bytylphenol were identified as markers for hill honey. Especially, for mountain honey, the markers were 4*H*-pyran-4-one-2,3-dihydro-3,5-dihydro-3,5-dihydroxy-6-methyl, 5-hydroxy-methyl-furfural, and 2,2,4-trimethyl-1,3-pentanediol diisobutyrate, and for urban honey, the markers were 4*H*-pyran-4-one-2,3-dihydro-3,5-dihydro-3,5-dihydroxy-6-methyl, 5-hydroxy-methyl-furfural, 2,2,4-trimethyl-1, and 3-pentanediol diisobutyrate.

Several studies of honey volatile composition that used SPME-GC-MS with chemometrics suggested that their combined usage in order to determine geographical origin of honey is a robust and reliable method of a high predictive ratio.

Ten chemical subclasses (sulphur compounds, ketones, aldehydes, alcohols, esters, nitrate compounds, aliphatic hydrocarbons, ethers, carboxylic and aromatic acids) of volatile compounds were identified for acacia honey samples from different geographical zones of Romania. In particular, the dominant compounds of acacia honey samples were 3-methyl-3-buten-1-ol for Transylvania region, ethanol, acetic acid, 5-ethenyldihydro-5-furanone for the southern part of Romania, acetone, 3-methyl-3-buten-1-ol, *trans*-linalool oxide, and benzemethanol for the eastern part of Romania. Applying statistical analysis, it was suggested that multiple volatiles are more suitable for discrimination of acacia honey based on geographical origin [2].

Another HS-SPME-GC-MS-based profiling for discrimination of citrus and thyme honey from different geographical origins of Greece was used by Aliferis and co-workers [47]. This variable classification was revealed by applying chemometric models, OPLS-DA, and OPLS-HCA, providing good discriminative ability. This discrimination was achieved using the most influencing fragments (*m*/*z*), without the identification of each compound.

Volatile compounds of Sudanese honeys were determined and included aldehydes, alcohols, carboxylic acids, ketones, esters, hydrocarbons, norisoprenoids, phenols terpenes, and derivatives. PCA was used and showed clear classification of the tested unifloral honeys with the same floral source from different geographical origin [78].

**Figure 2:**
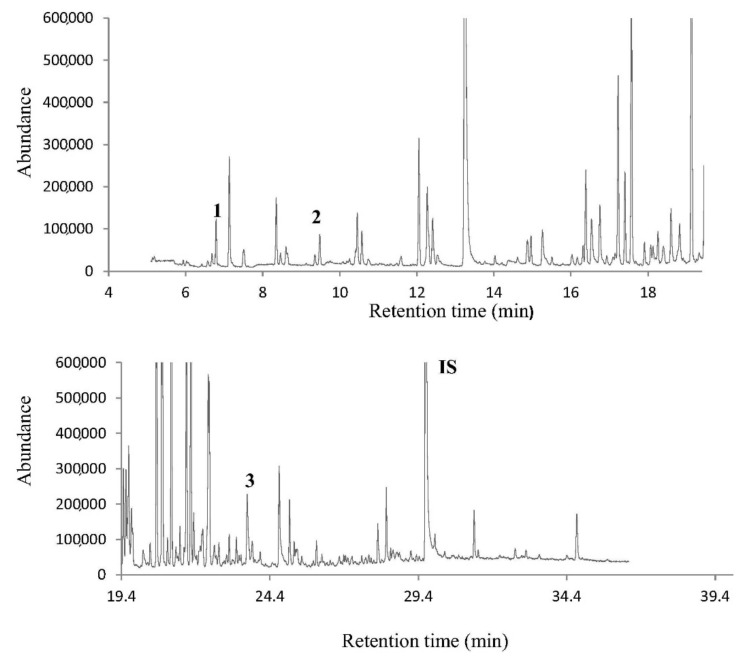
A typical gas chromatogram of citrus honey from Egypt. Possible volatile markers of geographical origin are indicated in bold. 1: heptane, 2: 2-methyl-butanal, 3: methyl anthranilate. IS: internal standard. Reprinted with permission from ref. [48]. Copyright 2016 Copyright Elsevier Ltd.

Determination of volatile profile of citrus honeys from different Mediterranean countries (Greece, Spain, Egypt, and Morocco) was performed using HS-SPME-GC-MS. LDA analysis of geographical sources of citrus honeys correctly classified these samples from different Mediterranean countries (Figure 2) [48]. Applying the above combination of techniques on Greek *Thymus capitatus* (L.) [61] and Greek pine [62] honey revealed that selected volatile compounds can clearly distinguish the geographical origin of these honeys. Performing MANOVA at the thyme honey samples, the volatile compounds of formic acid ethyl ester, formic acid, acetic acid, 1-hydroxy-2-propanone, octane, terpinen-4-ol, decanal, decanoic acid ethyl ester, and 4,7,7-Trimethyl-bicyclo (3,3,0)-octan-2-one were defined as markers for the determination of their geographical origin. In the case of pine honey samples as chemical markers of geographical origin, the following compounds were identified: hexanoic acid ethyl ester, 2,3-butanediol, decane, *β*-thujone, heptanoic acid ethyl ester, 1-methyl-4-(1-methylethenyl), benzene, nonanal, and 2-ethyl-1-hexanol.

Regarding the aforementioned studies, SPME-GC-MS fingerprinting has been proven to be a powerful method for the discrimination and classification of honey. Volatile markers of honey that determine its botanical and geographical origin include different classes of compounds, including monoterpenes, norisoprenoids, sesquiterpenes, benzenoids, alcohols, esters, ketones, and aldehydes, that have been proposed using also chemometric analyses potentiated the effectiveness of the above method. The assessment of botanical and geographical origin of honeys is based on the relative abundance of volatile compounds since their nature and relative amount represent a distinctive fingerprint.

The main source of volatile compounds of honey is the nectar of the flower, thus the monofloral honeys have a characteristic pattern of volatiles composition, and only these specific volatile compounds could be used for floral origin differentiation. However, these biomarkers are not always the same since differences—even within a single type of monofloral honey due to the plant variety, the geographical origin or local beekeeping practices—are often observed.

As mentioned above, a standard volatile profile of honeys is not possible since the chemical composition of honey is season-dependent and strongly affected by its geographic origin. Honey produced in different regions, areas, cities, and countries possesses a characteristic profile due to the climatic conditions. Moreover, the resulted volatile profile of honey is heavily dependent on the conditions of isolation and detection techniques. For example, regarding SPME, when the extraction is performed at higher temperatures, the conditions become favorable for the extraction of compounds with low volatility, and vice versa.

## 3. Authentication of Honey Using IR Spectroscopy

Infrared spectroscopy is considered an ideal technique for qualitative and quantitative determination of organic compounds. Specific absorption bands generated by characteristic groups of the compounds presented at a sample allow quality control of honey. The use of IR is widely accepted since it is low-cost, not sample-destructive, and easy to use. Moreover, analysis of the vast data obtained with chemometrics provide reliable results.

### 3.1. Detection of Honey Adulteration Using IR Spectroscopy

Infrared-based spectroscopy can be used for the detection of different adulterants in honey at different ranges of absorption. Chemometrics has been used as an essential tool for chemical fingerprinting of honey (Table 2).

Chen et al. [91] used near-infrared (NIR) spectroscopy on blossom honey to determine adulteration with high fructose corn syrups. The characteristic bands of blossom honey spectrum were around 6851 cm^−1^ (O–H stretch), 5607 cm^−1^ (CH_2_ group), 5201 cm^−1^ (O–H stretch and bend band), 4782 cm^−1^ (O–H deformation band and C–O stretch band), 4686 cm^−1^ (C–H stretch and deformation band), and 4182 cm^−1^ (CH_2_ stretch and deformation band). By using discriminant partial least squares analysis at different spectral ranges, it was shown that the adulterated honey could be better distinguished from unadulterated honey, with a correct classification rate of 92.13%, between 6000–10,000 cm^−1^. For the determination of high fructose corn syrup in honey, Ferreiro-González et al. [55] applied visible (Vis)-NIR spectroscopy. According to their results, HCA and PCA did not achieve full differentiation of the samples; however, honey samples were fully distinguished by using supervised LDA. The coefficients of the wavelengths 444.5 and 1462 nm are high and negative at low ratios of adulteration whereas at higher ratios of adulteration, they become positive. At 472.5 nm, the increase in adulteration leads to an increased negative coefficient. In another study, Fourier transform infrared (FTIR) spectroscopy was used to quantify corn syrup in honey to detect the adulteration based on sugar content. The differentiation between pure and adulterated honeys was obtained clearly at the spectral range of 1150–650 cm^−1^, which was characteristic of pure honeys [92]. Moreover, in a study by Li et al. [57], mid-infrared (MIR) spectroscopy combined with chemometrics (PLS) successfully quantified high fructose syrup (HFGS) in honey samples. The absorption maxima of pure honey and HFGS were achieved at 3285, 2930, 1642, 1370–1420, 1200–1350, and 1025 cm^−1^. The characteristic band at 3285 cm^−1^ (OH– stretching vibrations of water) raises by increasing HFGS concentration due to its high moisture. The authors fused the data provided by MIR and Raman spectroscopy at low-level, mid-level, and high-level to investigate the best model in terms of prediction accuracy of the detection of adulteration. After data fusion combined with chemometrics, the best prediction ability and stronger stability was revealed by the high-level model, compelling it ideal for quantitative analysis.

NIR spectroscopy combined with the competitive adaptive reweighted sampling (CARS)-PLS-LDA model seem to be effective to classify honeys in both cases of adulteration, with high fructose corn and maltose syrup. The characteristic peaks of absorbance were the same for pure and adulterated honeys: 6891, 5619, 5155, 4778, 4395, and 4231 cm^−1^. For the CARS-PLS-LDA analysis, the chosen variables were located in the ranges of 10,000–7300 cm^−1^, 6800–5500 cm^−1^, 4800–4200 cm^−1^ and 9800–8200 cm^−1^, 5620–5500 cm^−1^, and 4200–4100 cm^−1^ for each model. The spectral data were further statistically processed by using PLS regression and showed that the quantification was sufficiently obtained for maltose syrup-adulterated honey samples from same and different floral origins but was not obtained for high fructose corn syrup-adulterated honeys [93]. The same spectroscopic technique (NIR), using three different NIR instruments (a laboratory, as well as a portable and a mobile instruments), and statistical techniques (PLS-DA), was applied to South African honeys. Particularly good classification accuracies were obtained between the non-adulterated and adulterated honeys and verified the capability of NIR spectroscopy to detect the addition of sugars and cheap imported honey, irrespective of the type of instrument. Specifically, the PLS-DA model built on the data collected from the laboratory instrument shows that a significant contribution to the model is given by the intervals 1000.0–1038.6 nm, 1097.5–1132.8 nm, 1167.1–1199.6 nm, 1274.2–1283.4 nm, 1328.4–1355.0 nm, and 1375.9–1386.6 nm. In the case of the PLS-DA model from the portable instrument, the meaningful spectral intervals were 908.1–976.2 nm, 1143.5–1162.1 nm, 1205.4–1267.4 nm, and 1447.0–1453.2 nm. The last PLS-DA model from the mobile instrument indicated that the spectral regions which contribute most significantly to the discrimination among the categories are 861.8–888.8 nm, 955.9–989.3 nm, 1356.7–1363.1 nm, 1504.9–1530.6 nm, 1594.7–1754.4 nm, 1786.3–1856.5 nm, 2003.5–2035.4 nm, 2131.6–2196.0 nm, 2234.6–2273.4 nm, and 2423.0–2514.8 nm [65]. NIR spectroscopy combined with aquaphotomics were used to detect adulterants (corn, sucrose, high fructose, beet, and rice syrups) in Manuka honey. PCA and PLSR model regression vector analyses were performed at the spectral region of 1300–1800 nm, and 12 characteristic bands (1324, 1344, 1356, 1386, 1418, 1426, 1434, 1460, 1476, 1502, 1528, and 1586 nm) were selected according to the results of analysis, providing classification of non- and syrup-adulterated Manuka honeys [94].

**Figure 3:**
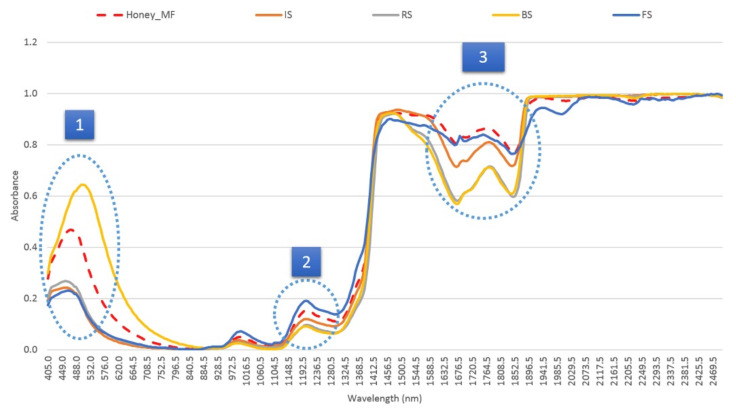
Average Vis-NIR spectra for pure multi-floral honey and for the four different sweeteners used for the adulteration (rice syrup (RS), invert sugar (IS), brown cane sugar (BS) and fructose syrup (FS)), in the regions 1, 2 and 3 the honey samples show different intensities depending on the type of adulterant. Reprinted with permission from ref. [50]. Copyright 2019 Copyright Elsevier B.V.

Aliaño-González et al. [50] used Vis-NIR spectroscopy combined with chemometrics (HCA, LDA, PLS) in order to guarantee the quality of multi-floral Granada Protected Designation of Origin (PDO) honey by determining common adulterants (rice and fructose syrups, invert and brown cane sugars). Thirteen significant wavelengths (465.5 nm, 499.0 nm, 559.5 nm, 675.5 nm, 736.0 nm, 1104.5 nm, 1170.5 nm, 1253.0 nm, 1324.5 nm, 1423.5 nm, 1467.5 nm, 1544.5 nm, and 1958.0 nm) were selected for the discrimination using Fisher’s linear discriminant functions. Most of the bands are characteristic regions (550–600 nm, 1190 nm, and 1700–1900 nm) of the Vis-NIR spectra (Figure 3). The combination of these techniques clearly distinguished non-adulterated from adulterated honeys, by developing an adulteration model which included all the adulterants used as well as a model for each adulterant.

In another study, FT-MIR technique was employed to support electrical impedance spectroscopy (EIS) analysis to characterize and quantify sugar adulterated honeys from different varieties. However, FT-MIR technique used alone successfully differentiated non-adulterated and adulterated honeys. Specifically, the addition of sucrose syrup was detected by the increase in absorbance in the region of 1800–650 cm^−1^ and the Full-Width-at-Half-Maximum (FWHM) was found at 1056 cm^−1^ for all honey samples, related to C–O, C–C, and O–H stretching, and was increased by increasing the concentration of the adulterant [95]. FT-MIR analysis was also carried out for pure and adulterated *Trigona* spp. and *Apis* spp. honey by Mail et al. [96]. The characteristic peaks of *Trigona* spp. and *Apis* spp. honey were 3272, 2934, 1643, 1416, 1345, 1256, and 1026 cm^−1^. In the case of Apis honey, the characteristic spectra were changed in all the regions with the addition of vinegar, even at low percentage due to the dilution by the amounts of water in the vinegar. The adulterated *Trigona* spp. honeys with water also shift away from pure honey at most of the spectral regions. Thus, the spectroscopic data showed that this technique could rapidly detect the adulterants in both honey types.

Attenuated total reflectance (ATR)-FTIR spectroscopy coupled with chemometrics was used in a study on stingless bee (*Heterotrigona itama*) honey from Malaysia for its capacity to detect adulteration by five adulterants including fructose, glucose, sucrose, corn syrup, and cane sugar. Applying PCA, all the adulterants were discriminated at the spectral region 1180–750 cm^−1^. Especially, the absorption peaks at 1054, 876, and 779 cm^−1^ were attributed to the increasing percentages of fructose. The characteristic peaks at 1022, 991, and 898 cm^−1^ were assigned to the presence of glucose, and at 991 and 921 cm^−1^ to the presence of sucrose. PLSR analysis was also able to quantity honey adulteration in all five cases [97]. In another study of honey adulteration with sugar, FTIR spectrometer with an ATR device was applied to honeys produced in different places of Ecuador combined with PCA. This combination showed to be ideal for the quality control of honey [59]. The ATR-FTIR technique has been also used alongside chemometrics for the estimation of the adulteration with commercial sugars of aren (*Arenga pinnata*), coconut, and cane sugar of Indonesian honeys. PCA and PLS analyses were applied for differentiation and quantification of the samples, respectively. It was proved that this combination is suitable for the detection of adulteration and measurement of the added sugar at Indonesian honeys [98].

Pure (105 samples) and adulterant (154 samples) honeys were analyzed by NIR and MIR spectroscopies combined with chemometrics to detect adulteration by rice and corn syrups. Principal components analysis (PCA) and PLS-DA models were used for adulterant determination. PCA was not able to distinguish adulterated honeys. However, clear discrimination of honey adulteration by spectrum data was shown by pretreatment of second derivative and by PLS-DA [80]. In another study, natural and syrup-adulterated honeys from China were analyzed using both spectroscopies, NIR and ATR-FTIR. Two types of adulterants were studied: type 1, rice and beet syrup, and type 2, high fructose corn, corn, maltose, and sucrose syrup. Between NIR and ATR-FTIR, more characteristic peaks were observed in the second technique. The spectral region at 750–1500 cm^−1^ was related to the absorption of major monosaccharides (such as fructose and glucose) and disaccharides (such as sucrose) and the region at 750–900 cm^−1^ was attributed to anomalous peaks corresponding to the characteristic absorptions of sugars. The integrated spectral data of honeys were subjected to Support Vector Machine (SVM) to detect adulteration. Data fusion and parameter optimization algorithms helped to create the best SVM model characterized by accuracy, sensitivity, and specificity in adulteration with syrup [51].

**Table 2:** Application of vibrational spectroscopic techniques coupled with chemometrics in detection of honey adulteration.

Type of Spectroscopy	Chemometrics Methods	Type of Adulterants	References
ATR-FTIR	PCA, SIMCA, PLS	Fructose syrup, glucose syrup, sucrose syrup, corn syrup, cane sugar	[97]
ATR-FTIR	PCA, DA, PLS	Commercial sugars of aren (*Arenga pinnata*), coconut, cane sugar	[98]
ATR-FTIR and Raman	PCA	Sucrose, reducing sugars	[59]
MIR and Raman	PLS, Data fusion	High fructose corn syrup, maltose syrup	[57]
NIR	DPLS	High fructose corn syrup	[91]
NIR	CARS, PLS- LDA	High fructose corn syrup	[93]
NIR	PLS-DA	Glucose syrup, fructose syrup, cheap imported honey	[65]
NIR	PCA, PLS	Corn syrup, sucrose syrup, high fructose corn syrup, beet syrup, rice syrup	[94]
NIR and MIR	PCA, PLS, DA	Rice syrup, corn syrup	[80]
NIR and ATR-FIIR	SVM, Data fusion	Type 1: rice and beet syrup, type 2: high fructose corn syrup, corn syrup, maltose syrup, sucrose syrup	[51]
Raman	PCA, PLS, artificial neural network ANN	Glucose, fructose, sucrose, maltose	[58]
Raman	Adaptive iteratively reweighted penalized least squares airPLS, PLS, DA	High fructose corn syrup, maltose syrup	[64]
Raman	SIMCA	Molasses, date molasses, grape molasses, high fructose corn syrup, corn syrup (dark and light), sucrose, inverted sugar	[63]
NIR	HCA, PCA, LDA, PLS	High fructose corn syrup	[55]
NIR	HCA, LDA, PLS	Inverted sugar, rice syrup, brown cane sugar, fructose syrup	[50]

### 3.2. Determination of Honey Origin Using IR Spectroscopy

The combination of IR with chemometrics provide satisfactory discrimination and rapid first-line classification of honey based on the botanical and geographical origin.

**Figure 4:**
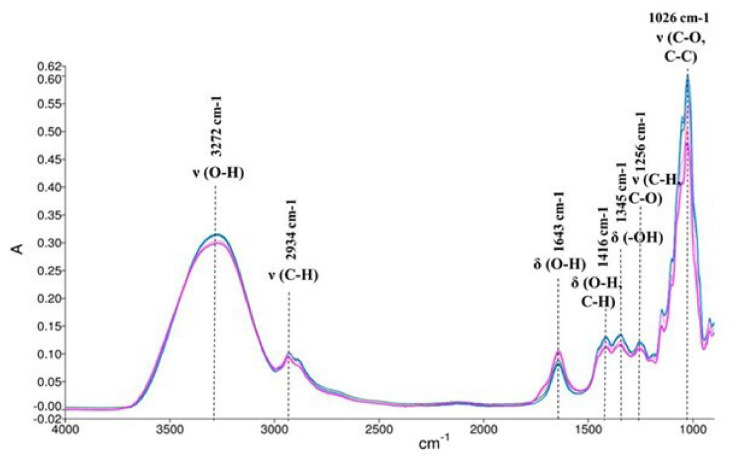
FTIR spectra curve and identified functional group region for Trigona honey (pink) and Apis honey (blue), x-axis: wavelength and y-axis: Absorbance. Reprinted with permission from ref. [96]. Copyright 2019 Copyright Oriental Scientific Publishing Company.

A study by Mail et al. [96] suggested that *Trigona* spp. honey can be clearly distinguished from *Apis* spp. honey using FTIR technique, based on the differentiation of their absorbance at the identified functional group regions. Both honeys presented almost the same spectra, but *Trigona* spp. honey exhibited lower absorption at the region of carbohydrates (3280–3271 cm^−1^, 2935–2931 cm^−1^, 1416–1252 cm^−1^, 1031–1020 cm^−1^) and higher absorption at region of water (1643–1642 cm^−1^) in comparison with *Apis* spp. honey (Figure 4). Similarly, the botanical source of 30 honey types of eight different varieties (eucalyptus, litchi, neem, lemon, ginger, Kasmiri white, BR Hills, and Pan India) was also evaluated by ATR-FTIR spectroscopy and chemometrics. PCA was employed and successfully classified the honey samples, based on the spectral differences in the region of 1800–750 cm^−1^. Through the comparative overlay of ATR-FTIR spectra of the different honeys in this region, the characteristic peaks were 1636, 1454, 1431, 1366, 1261, 1151, 1104, 1079, 1057, 1034, 967, 926, and 887 cm^−1^ [9]. Seventy Italian honey samples from seven different botanical sources (acacia, orange, chestnut, eucalyptus, lavender, honeydew, and linden) were analyzed using FT-NIR spectroscopy. The spectroscopic data were further evaluated by PLS-DA and by sequential and orthogonalized covariance selection (SO-CovSel)-LDA. According to Variable Importance in Projection (VIP), NIR spectroscopy did not achieve particularly good classification between the seven different varieties of honey samples. However, the mid-level data approach performed a more accurate prediction of the honey samples belonging to the alternative class “Others”, providing fewer false positives than the other strategies and improvement in the overall classification rates [99].

Near-infrared spectrum and mid-infrared spectrum of three different raw honey sources (vitex, jujube, and acacia) have been collected to evaluate their botanical origin. According to spectral data and by using different types of chemometric analysis models, PLS-DA, SVM, and interval partial least squares (iPLS), a rapid and accurate classification of honeys based on their botanical origin was achieved. By using the iPLS model, it was revealed that the optimized spectral regions for botanical discrimination of NIR were 6310–5847 cm^−1^ and of MIR were 3397–3298 cm^−1^, 2893–2592 cm^−1^, and 1381–980 cm^−1^ [80].

The combination of IR spectroscopy with other techniques was also reported for botanical characterization in some studies.

For botanical characterization of eight different varieties of Italian honeys, five different analytical techniques (IR, NIR and Raman spectroscopy, Proton Transfer Reaction-Mass Spectrometry (PTR-MS), and electronic noise) and fused data by PLS-DA were used. The analysis of the regression coefficients showed the effectiveness of NIR spectroscopy to discriminate almost all the investigated classes. Specifically, the band at 4000–4180 cm^−1^ was characteristic for chestnut, sunflower, and multiflower honeys, while the band at 4180–4230 cm^−1^ was observed at citrus, linden, chestnut, and rhododendron samples. Sunflower, multiflower, and chestnut honeys were differentiated by other honey samples at 4232–4296 cm^−1^. Discrimination of citrus, linden, and chestnut from honeydew, robinia, and sunflower was performed at 4296–4388 cm^−1^; for citrus, linden, rhododendron from robinia, multiflower, honeydew, and chestnut, discrimination was performed at the band 4388–4590 cm^−1^. Moreover, the combination of Raman and NIR spectroscopy and PTR-MS provided the best results of honey samples discrimination based on botanical origin, as verified by PLS-DA and high-level data fusion method [16]. Different physicochemical techniques such as elemental profiling, stable isotope analysis, metabolomics, quadrupole time of flight mass spectrometry (UPLC-QToF MS), and NIR, FT-IR, and Raman spectroscopic fingerprinting were used for botanical discrimination of four different honeys (rata, kamahi, clover, and manuka) from New Zealand by using also multivariate statistical analysis. OPLS-DA was applied to evaluate the best technique for classification or to prove whether their combination provides more accurate results. The best discrimination of honeys was achieved by metabolomic and element/isotopic data. In the case of spectroscopic techniques (NIR, FT-IR, and Raman), the best results were obtained in combination with the other techniques for floral classification of honeys [56].

For geographical discrimination in a study by Guelpa et al. [65], near-infrared spectroscopy coupled with statistical analysis was performed to identify authenticity of South African honey. By applying PLS-DA on the spectrum data, honeys were successfully classified based on the geographical origin—South African honeys were differentiated from non-South African honeys. The characteristic bands concerning the geographical classification were shown at the above paragraph 3.1, related also with the determination of adulteration. Differentiation of wild honeys from different areas of Indonesia was achieved using ATR-FTIR technique coupled with multivariate statistical analysis. The spectroscopic data were subjected to DA for discrimination of honeys. The best discrimination model was obtained at the wavenumbers of 327, 1110, and 2933 cm^−1^ [98].

As far as the NIR region is concerned, the spectral curves and the absorbance peaks were similar for pure and adulterated honey. The characteristic peaks are 6851, 5618, 5157, 4762, 4394, and 4103 cm^−1^. The absorption peak at 6851 cm^−1^ corresponds to first overtone of the O–H stretch. The peak at 5618 cm^−1^ is the second harmonic of C=O absorption, while the peak at 5157 cm^−1^ is assigned to the overtone of O–H stretching and bending. The peak around 4762 cm^−1^ belongs to a combination of O–H deformation band and C–O stretch band. The peak at 4394 cm^−1^ is ascribable to the overtone of C–H stretching and deformation and at 4103 cm^−1^ to the combination of CH_2_ stretch and deformation band. However, there are important peaks around 8403, 6839, 5168, 4773, 4386, and 4231 cm^−1^, where the samples show different intensities depending on the type of the adulterant (sucrose, fructose, and glucose).

In general, the MIR spectra of honey consist of six characteristic regions at specific wavenumbers, 3285, 2930, 1642, 1370–1420, 1200–1350, and 1025 cm^−1^. The absorption peak at 3285 and 1642 cm^−1^ are assigned to the O–H stretching and bending vibrations of water, respectively. The absorption maxima at 2930 cm^−1^ corresponds to stretching vibrations of O–H and the bands at 1370–1420 cm^−1^ correspond to deformation vibration of C–H from cellulose and lipids. The peaks around 1200–1350 cm^−1^ are N–H deformation and C–N stretching vibrations. The absorption peak at 1025 cm^−1^ is assigned to C–O and C–H stretching vibrations. For the detection of adulteration, there are some absorption peaks at the region of 750–1500 cm^−1^ originating due to monosaccharides (such as fructose and glucose) and disaccharides (such as sucrose) in honey. In particular, the region 700–950 cm^−1^ is known as the “anomeric region of carbohydrates”, contains anomalous peaks corresponding to the characteristic absorptions of sugars. In the case of adulteration of honey with high fructose syrup, the absorption peaks at 3285 cm^−1^ are increased due to the high moisture content. Moreover, major differentiation in the MIR spectra of adulterated honey is observed at 1054, 867, 822, and 779 cm^−1^ by the presence of fructose, at 1022, 991, and 898 cm^−1^ by the presence of glucose and at 991 and 921 cm^−1^ by the presence of sucrose. Even though pure and adulterated honey provide similar spectra, the different absorption intensities at the characteristic peaks make it possible to use NIR and MIR for their identification.

It seems that the variation of honey associated with botanical and geographical origin using NIR and MIR spectroscopy is mainly based at 4000–8000 cm^−1^ and 800–4000 cm^−1^, respectively. In both cases, the optimized region of discrimination dependent on the chemometric model. Thus, depending on the discriminant model used, wavelengths corresponding to the vibrational transition of the main functional groups allow for sufficient variation.

## 4. Authentication of Honey Using Raman Spectroscopy

Raman spectroscopy is a suitable, efficient, fast, and inexpensive technique for quality control and evaluation of the chemical properties of honey. The main advantages of Raman spectroscopy are the small amount of sample required, the speed of analysis, the high reproducibility of data, and the avoidance of interference related to the water molecule. The verification of the origin and control of the authenticity of honey can be facilitated using chemometric approaches.

### 4.1. Detection of Honey Adulteration Using Raman Spectroscopy

Raman spectroscopy can be successfully used to detect adulteration of honey (Table 2).

Raman technique coupled with multivariate analysis was applied at honeys to identify and quantify sugars (glucose, fructose, maltose, and sucrose contents) and further to characterize them as adulterants. The characteristic spectral bands that correlated to sugars of honey were 314, 341, 415, 530, 617, 744, 776, 790, 838, 856, 911, 933, 1028, and 1106 cm^−1^. PCA, partial least squares (PLS), and artificial neural network (ANN) were used to extract differentiation from the spectroscopic data which successfully led to the discrimination of sugar contents in honey [58]. Moreover, Raman technique was used by Salvador et al. [59] to detect the sugar content and the type of adulteration in commercial honeys of Ecuador. The main observed bands of honeys from Pichincha and Loja provinces were 326, 338, 419, 516, 630, 707, 817, 862, 918, 1062, and 1126 cm^−1^. These bands were assigned to the presence of sugar (glucose, fructose, and sucrose) at honey samples. The bands of pure honey at 817 and 862 cm^−1^, in the case of adulteration with sucrose, were overlapped with strong absorptions at 822 and 834 cm^−1^. Principal component analysis was applied and confirmed the applicability of Raman technique for the detection of adulteration of honey with glucose, fructose, and sucrose.

**Figure 5:**
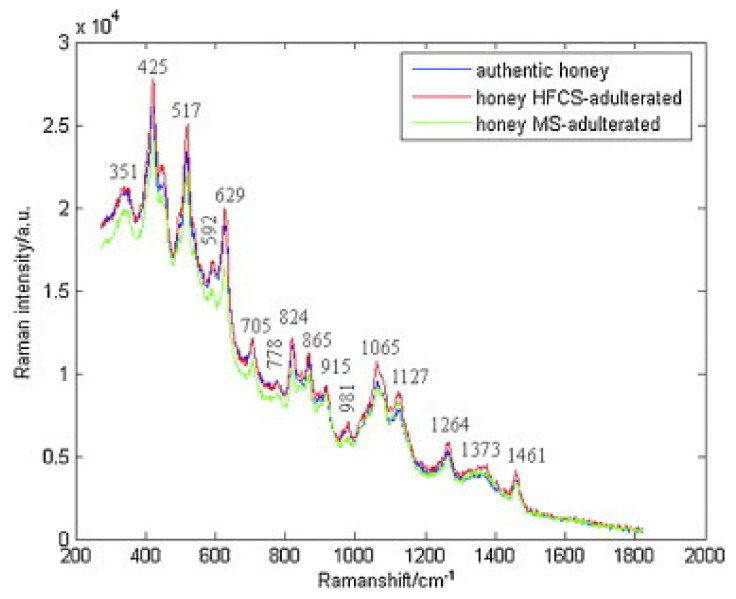
Raman spectra of a randomly selected authentic honey sample and the same honey sample adulterated with high fructose corn syrup (40%, *w*/*w*) and maltose syrup (40%, *w*/*w*). Reprinted with permission from ref. [64]. Copyright 2012 Copyright Elsevier Inc.

In another study, Raman spectroscopy was also used to detect adulteration of honey with high fructose corn syrup and/or maltose syrup. The characteristic bands corresponding to authentic and adulterated honeys were observed: 351, 425, 517, 592, 629, 705, 778, 824, 865, 915, 981, 1065, 1127, 1264, 1373, and 1461 cm^−1^ (Figure 5). The spectra data were subjected to adaptive iteratively reweighted penalized least squares (airPLS). Using PLS-LDA, classification of honeys was achieved in both cases of adulterants and in mixtures of them [64]. Chemometrics with Raman spectroscopy were successfully employed for the quantification of HFGCS (high fructose syrup) in adulterated honey, as well. At the band of 2791 cm^−1^, the absorption was increased by increasing the HFGS concentration, while at 1130 cm^−1^, the absorption was reduced due to the decrease in protein and amino acid content in the adulterated honeys. Three data fusion strategies were used and showed high predictability in the adulteration of honey, while the best results were obtained by the high-level data fusion process [57].

Non-invasive techniques using a handheld and compact benchtop Raman system were employed to detect honey adulteration by molasses, date molasses, grape molasses, high fructose corn syrup, corn syrup (dark and light), sucrose, and inverted sugar. The characteristic spectroscopic bands found at 424, 517, 629, 706, 824, 1067, 1127, 1265, 1373, and 1461 cm^−1^ were concerning the presence of sugars. By performing SIMCA, classification of the pure and adulterated honeys with 100% specificity and sensitivity was achieved [63].

### 4.2. Detection of Honey Origin Using Raman Spectroscopy

Raman technique is capable of on-site testing of honey samples to authenticate and verify their label information based on its origin.

**Figure 6:**
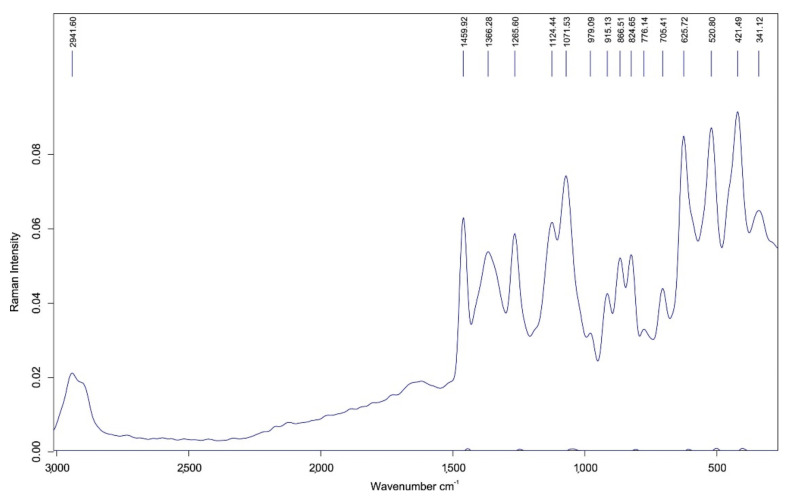
Average FT-Raman spectrum of the *Lavandula* spp. honey. with permission from ref. [54]. Copyright 2017 Copyright Elsevier B.V.

An analysis of one hundred *Lavandula* spp. honeys from different regions of Portugal was performed by FT-Raman spectroscopy combined with chemometrics (PLS) to determine their chemical composition. *Lavandula* spp. honey showed characteristic peaks at the region of 200–1500 cm^−1^. Specifically, the characteristic spectral peaks were found at 341, 422, 521, 626, 705, 776, 825, 867, 915, 979, 1072, 1124, 1266, 1366, and 1460 cm^−1^. The combination of the above techniques could be considered as a reliable tool for the quality prediction of *Lavadula* spp. honey (Figure 6) [54]. FT-Raman spectroscopy and statistical analysis were applied at commercial honeys to authenticate their labeling. According to the spectroscopic data generated (424, 517, 629, 706, 824, 1067, 1127, 1265, 1373, and 1461 cm^−1^) and based on SIMCA analysis verification, the prediction of pure honey samples was rapidly and efficiently achieved [63].

Four pure honeys with different floral origins (clover, kamahi, manuka and rata) obtained from producers in New Zealand were discriminated using Raman spectroscopy. According to OPLS-DA, the optimum honey discrimination was achieved with the combination of NIR, FT-IR, and Raman techniques with elemental profiling, stable isotope analysis, and metabolomics [55]. In another study, Raman spectroscopy and other analytical techniques (IR and NIR spectroscopy, PTR-MS, and electronic noise) combined with multivariate data fusion methods were used for botanical discrimination of eight different varieties (citrus, chestnut, linden, sunflower, honeydew, multiflower, robinia, and rhododendron) of honey from Italy. Data analysis shows that chestnut and linden honeys were discriminated more effectively from the others, characterized by the largest positive or negative coefficient. In the case of chestnut honey, the region at 200–500 cm^−1^ and the band at 524 cm^−1^ allowed its discrimination from the other types. The discrimination of linden honey was achieved due to the bands at 1492, 1576, and 1666 cm^−1^ [16].

The Raman spectra of honey shows most of the spectral peaks in the region between 200–1500 cm^−1^, thus, the differentiation of authentic and non-authentic honey is obtained at this region. The characteristic bands generated from Raman spectra of honey, which were used to detect the presence of sugars as adulterants, were observed at 314, 341, 424, 517, 629, 706, 824, 871, 918, 979, 1067, 1127, 1265, 1373, and 1461 cm^−1^. Moreover, at these spectral peaks the main differences for botanical and geographical discrimination were also revealed using chemometric analysis.

In particular, the region from 200–500 cm^−1^ is assigned to skeletal vibrational modes, namely C–C–C–, C–C–O, C–O, and C–C. The peaks 424 and 517 cm^−1^ correspond to deformations of C–C–O and C–C–C, the peak at 629 cm^−1^ is assigned to ring deformations of fructose, while the peak at 706 cm^−1^ corresponds to the stretching of C–O and bending vibrations of C–C–O and O–C–O of glucose. The spectral peak at 776 cm^−1^ was assigned to the C–C stretching and C–H vibrations present in glucose. The two peaks at 825 and 871 cm^−1^ are related to the vibration of C–H and CH_2_ deformation and C–O–H bending of fructose. The signals around 918 and 979 cm^−1^ are assigned to vibrations of C–H and C–O–H, and to two anomers of fructose and glucose, respectively. The peak around 1067 cm^−1^ is due to the C–H and C–O–H bending of carbohydrates and due to a minor contribution of vibration of C–N bonds in amino acids and proteins. The peak around 1127 cm^−1^ is a combination of stretching vibration of C–O and C–O–C and vibration of C–N of proteins and amino acids. This peak is also related to deformation of C–OH of glucose and sucrose. The signal at 1265 cm^−1^ is related to C–O–H, C–C–H, and O–C–H vibrations, and for fructose it is related to C–O–C cyclic alkyl ethers. The band at 1373 cm^−1^ is assigned to the bending of C–H and O–H bonds, also for glucose and sucrose. Finally, the peak at 1461 cm^−1^ is related to the combination of the vibration of the COO group and the bending vibration of the CH_2_ group. This peak also is attributed to symmetric deformation mode of CH_2_ in fructose and the presence of flavanols and organic acids.

## 5. Conclusions

Honey consumption gradually raises mainly because of its health benefits. However, an important issue that consumers, producers, industries, and researchers must deal with is the verification of its authenticity in terms of botanical and geographic origin. Nonetheless, adulteration cases should not be neglected. Consequently, it is urgent to develop low-cost, simple, and reliable techniques that will ensure authenticity of honey. In this regard, SPME-GC-MS based on the volatile fraction was proved to provide reliable results able to determine the authenticity of honey as far as its botanical and geographical origin. Furthermore, spectroscopic methods, namely IR and Raman, can also evaluate both botanical and geographical origins of honey. Moreover, spectroscopic techniques were also able to detect adulteration, mainly with sugar syrups. However, to interpretate the complicated results, chemometric analysis was used. In general, the above-mentioned techniques combined with chemometric analysis are a powerful tool able to “screen” honey quality and to ensure consumers its authenticity. The present review contributes to the amplification and development of a methodology for the authenticity of honey, which will allow the market to verify the label description and the quality of the product.

## Figures and Tables

**Table 1:** Employment of SPME-GC-MS technique for the determination of volatile compounds for botanical characterization of unifloral honeys.

Floral Origin	Volatile Compounds as Markers for Botanical Source	Geographical Origin	References
Acacia	8-Hydroxylinalool, 2-furfural-aldehyde, *2*-hexen-1-ol, 2-hydroxycyclopent-2-en-1-one, 2-phenylethyl isothiocyanate, 2-phenylpropenal, 5-hydroxymethylfurfural, decanal, dimethyl palmitamine, hotreniol, lilac aldehyde C, lilac aldehyde D, linalool oxide, myrtenal, octanoic acid, oleic acid, pinocarvone	Romania	[8]
	Benzaldehyde, nonanal, phenylacetaldehyde	Poland	[37]
	(2*S*,2′*R*,5′*R*)- Lilac aldehyde B, (2*S*,2′*R*,5′*S*)-lilac aldehyde C, hotrienol	Slovakia, Czech Republic, Romania, Germany, Serbia, Georgia, Poland, Moldova	[88]
Buckwheat	Furfural, *2*-methylbutanoic acid, 3-methylbutanoic acid, 2-methylbutyraldehyde, 3-methylbutyraldehyde	Poland	[37]
	3-Methylbutanoic acid, 2-methylbutanal, phenylacetaldehyde	Italy, east Europe	[87]
	3-Methylbutanal, butanoic acid, 3-hydroxy-4,5-dimethyl-2(5*H*)-furanone, phenylacetaldehyde	Finland	[13]
Chestnut	*trans*-Linalool oxide, hotrienol, (*R*)-4-terpineol	Italy	[88]
	2-Aminoacetophenone, benzaldehyde, acetophenone, nonaic acid, octanoic acid, 3-furaldehyde	Corsica Island	[89]
Citrus	Lilac aldehyde, 1-*p*-menthen-9-al isomers, limonene, methyl anthranilate	Greece	[47]
	Linalool, *E*-linalool oxide, limonene	Greece	[48]
	4-Methoxy-benzaldehyde, lilac aldehydes A- D (isomers I-IV), *α*-4-dimethyl-3-cyclohexene-1-acetaldehyde, 2-cyclohexene-1-propanal, methylanthranilate, linalool, herboxide (isomer II), *cis*-linalool oxide, dill ether	Greece	[77]
*Citrus aurantium*	(2*S*,2′*R*,5′*R*)- Lilac aldehyde B, (2*S*,2′*R*,5′*S*)-lilac aldehyde C, (2*R*,2′*R*,5′*S*)-lilac aldehyde D	Greece, Italy, France	[88]
Fir	Ethyl hexanoate, ethyl heptanoate, ethyl octanoate, ethyl nonanoate, ethyl decanoate, ethyl dodecanoate, ethyl tetradecanoate, 6-methyl-5-hepten-2-one, 2-hydroxy-3,5,5-trimethyl- cyclohex-2-en-one, 1-(2-furanyl)-ethanone, nonane, (*Z*)-5-methyl-4-nonene, 3,4,5-trimethyl-phenol, nonanal	Greece	[60]
	Nonanal	Greece	[77]
Heather	3,4,5-Trimethylphenol, phenylic acid, benzoic acid, *β*-damascenone, isophorone	Poland	[37]
	Isophorone, 2-methylbutyric acid	Estonia	[38]
	Hotrienol	Northwest of Iberian Peninsula (Spain, Portugal)	[90]
Linden	6-Allyl-o-cresol, allylphenylsulfide, butanoic acid, 3,6-dimethyl-4,5,6,7-tetrahydro-1-benzofuran, 1-methyl-4-(1-methylpropyl)-benzene, *trans*-dihydrocarvone, *o*-methylacetophenone, isoneral, isopropyl benzene, geranic acid, sabinene, teresantalol, 2-undecenal, *p*-cymene, myrtenyl acetate	Romania	[8]
	Dimethylstyrene	Poland	[37]
	4-Terpineol	Slovakia, Czech Republic, Romania, Hungary, Moldova	[88]
Pine	*β*-Thujone, octane	Greece	[60]
	2-Hydroxybenzaldehyde	Greece	[77]
Rapessed	3-Methylpentanol, pentanoic acid, ethyl 2-hydroxy-4-methylbenzoate, *trans*-linalool oxide, *α*,*α*,4- trimethylbenzyl alcohol, lilac alcohol C, 3-phenyl propanol, dihydro-5-propyl-2(3*H*)-furanone, ethyl decanoate, ethyl 3-hydroxytridecanoate, ethyl dodecanoate, 2,2,4-trimethyl-1,3-pentanediol diisobutyrate, ethyl palmitate, ethyl oleate, ethyl benzoate, 3-methylbutanol	Romania	[8]
	Hexanal*, p*-cymene, 4-methyloctane, cumene, 3-caren-2-ol, *β*-phellandrene, 4-methyl-2,7-octadiene, 2,6-dimethyl-3,5,7-octatriene, *trans*-sabinene hydrate, verbenone, 1,3,8-*p*-menthatriene, *p*-*sec*-butyltoluene, *o*-anisaldehyde, carvacrol	Lithuania	[12]
	Benzoic acid, benzyl alcohol	Poland	[37]
	(*R*)-Linalool	Slovakia	[88]
Sunflower	3-Furfural aldehyde, (3,3-dimethylcyclohexylidene) acetaldehyde, 1,3,3-trimethylcyclohex-1-ene-4-carboxaldehyde, *p*-menthan-3-one, endo-borneol, menthol, myrtenol, verbenone, isopiperitone, *p*-cymen-7-ol, eugenol, *β*-calarene, *cis*-linalool oxide(furanoid), hotrienol	Romania	[8]
	4-Terpineol, *trans*-linalool oxide	Slovakia, Ukraine	[88]
Thyme	Formic acid, hexadecanoic acid, 1-octanol, 1-hydroxy-2-propanone, decane	Greece	[60]
*Thymus capitatus*	1,3-Diphenyl-2-propanone, 1-butyl-3-methylbenzene, 3,4,5-trimethoxy benzaldehyde, 3,4-dimethoxy benzaldehyde, vanilline, thymol	Palestine	[86]
*Thymus capitatus*	Phenylacetaldehyde, 1-phenyl-2,3-butanedione, 3-hydroxy-4-phenyl-2-butanone, 3-hydroxy-1-phenyl-2-butanone, 3-hydroxy-4-phenyl-3-buten-2-one	Greece	[47]
*Thymus capitatus*	Pentanoic acid, phenylacetonitrile	Greece	[77]
Algaroba (*Prosopis juliflora* (Sw.) DC)	Rose oxide	Brazil	[71]
Angico (*Anadenanthera colubrina*)	Benzenethanol	Brazil	[71]
Caraway	Benzaldehyde	Lithuania	[12]
Chanana (*Turnera ulmifolia* L.)	D-Sylvestrene	Brazil	[71]
Christ’s thorn (*Paliurus spina-christi*)	Nonanal, lilac aldehyde (isomers I-IV), decana, methyl nonanoate, hexanoic acid, 2-ethylhexanoic acid	Croatia	
Cloudberry	1-Propanol, *p*-cymene, isophorone, citral	Finland	[13]
Honey-dew	2,3-Butanethiol, acetic acids isomers	Poland	[37]
Juazeiro (*Ziziphus juazeiro* Mart)	Aromatic aldehydes, benzaldehyde, benzeneacetaldehyde	Brazil	[21]
Jurema branca (*Mimosa arenosa* willd Poir)	Sulfur compounds, ketones, hexanol, limonene, *α*-farnesene, *δ*-cardinene	Brazil	[21]
Lacy phacelia	Hexan-1-ol, lavender lactone	Poland	[4]
Lingonberry	Vanillin, 3-hydroxy-4,5-dimethyl-2(5*H*)-furanone, ethyl 3-phenylpropanoate	Finland	[13]
Malicia (*Mimosa quadrivalvis* L.)	Linalool	Brazil	[71]
Sweetclover	Phenylc acetic acid, (*Z*)-3-nonenal	Finland	[13]
Thistle	Nonanal, furfural, decanal, 3,6-dimethyl- 2,3,3*a*,4,5,7*a*-hexahydrobenzofuran, benzaldehyde, *α*-linalool, lilac aldehyde (isomer IV), hotrienol, phenylacetaldehyde, 4-oxoisophorone, benzyl alcohol, 2-phenylethanol, octanoic acid, nonanoic acid, methyl anthranilate	Italy	[17]
*Thymelaea hirsuta*	Benzene propanol, benzylalcohol, hexanol, 4-methoxyphenol	Palestine	[86]
*Tolpis virgata*	3,5-Dihydroxytoluene, tridecane	Palestine	[86]
Ulmo	Benzaldehyde, octane, nonanal, 4-methoxybenzaldehyde, isophorone, *β*-damascenone, lyrame, 4-vinylanisole	Chile	[14]
Velame branco (*Croton heliotropiifolius* Kunth)	Volatile acids	Brazil	[21]
